# Hospital Sunday Fund

**Published:** 1909-07-31

**Authors:** 


### HOSPITAL ADMINISTRATION.

### CONSTRUCTION AND ECONOMICS.

### HOSPITAL SUNDAY FUND.

#### THE BATTERSEA ANTI-VIVISECTION HOSPITAL.

The Lord Mayor presided at the Mansion House on the 28th inst. over a meeting of the Council of the Metropolitan Hospital Sunday Fund. Among those present were Lord Cheylesmore, Lord Leith of Fyvie, Sir William Church, Sir Henry Burdett, Sir Savile Crossley, Sir Richard Martin, Sir John Bell, the Archdeacon of London, the Rev. E. H. Pearce, Sir Joseph Dimsdale, Prebendary Russell Wakefield, the Hon. Harry Lawson, the Rev. C. H. Grundy, and Sir Edmund Hay Currie (Secretary).

Sir William Church, in moving that the report be received and that the awards recommended be paid as soon as possible, pointed out that the list of awards included a hospital which had not been recommended before by the committee—namely, the Battersea General Hospital, better known as the Anti-Vivisection Hospital. He said that the reason why this institution had not appeared on the list in former years was that it was not founded and maintained with the sole object of relieving the sick and suffering poor of Battersea, but it was also founded and maintained for keeping prominently before the public the work of anti-vivisectors. The name of the hospital showed that it had some other object than the relief of the sick poor. Then, again, the regulation of the hospital was such that the committee had always thought they could not include it in their list; the members of the medical staff were narrowed down to those qualified men who believed, rightly or wrongly, that no improvement was likely to take place in medical treatment in the future, because the whole of the civilised world had agreed that it was from the scientific experiments on animals that the knowledge of diseases was to be increased. No self-respecting medical man would for one moment consent to the governing body of a hospital, which consisted mainly of laymen, dictating to him his treatment of patients, which greatly narrowed the choice of the medical staff. It was these points, together with the general tenor of the annual report of the hospital, which had caused the committee not to bring the institution before the Council. This year the committee had been approached by various members of the Council, including some of the clerical members, who had testified to the great appreciation which the poor of Battersea had of the benefits which they received from this hospital. The committee, therefore, while disapproving, as much as ever, of the way in which the hospital was carried on, had brought the matter forward, and desired that the decision should rest rather with the Council than the committee. In the event of the Council's determining that a grant should be made, the committee desired to amend the grant and make it double the sum which was set down in the list—namely, £97 10s., in order that the grant should be made on the full basis.

Lord Leith, in seconding the adoption of the report, said that looking at the matter from the standpoint of broad policy, he felt that the action of the Distribution Committee would do good.

Mr. Thomas Bryant said he would always consider that the Anti-Vivisection Hospital cast a great slur upon the profession generally, and that they ought not to support a hospital which was based upon such principles. Some years ago, when associated with the Bolingbroke Hospital, it was his duty to see the surgery of that institution, and a great deal of it consisted of neglected and badly-treated cases from the so-called Anti-Vivisection Hospital. He moved as an amendment that the grant to the hospital in question be omitted.

The amendment found no seconder and therefore dropped.

Sir Henry Burdett said he shared Mr. Bryant's objections, and believed that the majority of the Council agreed with this view. At the same time, they had to consider the difficulties of the case, including those difficulties which the clergy experienced in dealing with the matter. They were making this grant not because they approved of the institution, or disapproved of it any less than they had ever done, but simply because it might, and probably did, do a measure of good among the people of Battersea. He hoped its authorities would purge themselves of the pretentious humbug involved in the claims they put forward as to the special advantages of treatment in that hospital from the anti-vivisectionist point of view, because the medical profession knew perfectly well that in the treatment of disease they must employ remedies which would not have been available had it not been for the discoveries resulting from vivisection. He certainly thought that, in giving the grant, it should go forth that each year they had the right, and would exercise it, of considering how this hospital was being administered. It was given in the hope that the authorities of this institution would mend their ways, purge their methods, and, in fact, fall into line with all the best-administered hospitals in the country.

The report was then adopted.

Archdeacon Sinclair moved a vote of thanks to the Distribution Committee.

The Rev. C. H. Grundy hoped it would be distinctly understood that the award of the grant gave the Council the right to express their opinion on the administration of this hospital, and that it would not be thought they had swallowed their principles after a certain amount of agitation.

The resolution was adopted.

Sir Joseph Dimsdale moved that the thanks of the Council be given to the editors of newspapers who had pleaded the needs of the hospitals and who had advocated the cause of the Fund. In doing so he paid a warm compliment to the London newspapers for the services they rendered in the cause of charity and benevolence, and remarked that in the Hon. Harry Lawson, whom he was glad to see present, they had the representative of one of the most charitable newspapers in existence. (Hear, hear.)

Sir Henry Burdett, in seconding the resolution, suggested that if the newspapers concentrated their assistance in the week immediately preceding Hospital Sunday a sum of £100,000 might be annually realised.

Cordial thanks were also given to the Committee of Distribution for their care in preparing the awards, and to the Lord Mayor for presiding.

